# Laparoscopic Surgery for Focal-Form Congenital Hyperinsulinism Located in Pancreatic Head

**DOI:** 10.3389/fped.2022.919238

**Published:** 2022-07-19

**Authors:** Zhe Wen, Jieqin Wang, Qifeng Liang, Xiaopan Chang, Wen Zhang, Huilin Niu, Qiao He

**Affiliations:** ^1^Department of Pediatric Surgery, Guangzhou Women and Children's Medical Center, Guangzhou Medical University, Guangzhou, China; ^2^Department of Genetics and Endocrinology, Guangzhou Women and Children's Medical Center, Guangzhou Medical University, Guangzhou, China; ^3^Department of Pathology, Guangzhou Women and Children's Medical Center, Guangzhou Medical University, Guangzhou, China; ^4^Department of Nuclear Medicine, The First Affiliated Hospital of Sun Yat-sen University, Guangzhou, China

**Keywords:** hyperinsulinism, laparoscopy, near-total pancreatic head resection, pancreaticojejunostomy, surgery

## Abstract

**Background and Aims:**

Congenital hyperinsulinism of infancy (CHI) is a rare condition that may cause irreversible severe neurological damage in infants. For children in whom medical management fails, partial or near-total pancreatectomy is then required according to the type of lesion. Currently, open surgery of near-total pancreatic head resection is a mature technique for the treatment of focal-form CHI located in the head of the pancreas, but a minimally invasive laparoscopic procedure has not been reported yet. The aim of this study was to verify the feasibility, safety, and efficacy of laparoscopic pancreatic head resection and Roux-en-Y pancreaticojejunostomy for focal-form CHI.

**Methods:**

Two infants with persistent hypoglycemia and increased insulin levels were diagnosed with CHI and underwent laparoscopic near-total pancreatic head resection due to a suboptimal response to medical therapy and the likelihood of focal disease amenable to surgery. Clinical records, operative findings, and postoperative follow-up were collected and analyzed.

**Results:**

The operative duration was 300–330 min, and the intraoperative blood loss was minimal. The duration of postoperative abdominal drainage was 4–5 days. Neither intra- nor postoperative abdominal complications occurred. Oral feeding was resumed 3–4 days after the operation, and the blood glucose level was gradually stabilized to within the normal range. Normal blood glucose was observed in both patients over a follow-up period of 3–6 months.

**Conclusions:**

Laparoscopic pancreatic head resection and Roux-en-Y pancreaticojejunostomy can be considered a safe and effective procedure with minimal morbidity and excellent outcomes for the treatment of focal CHI in the head of the pancreas.

## Introduction

Congenital hyperinsulinism of infancy (CHI) is the most common cause of persistent hypoglycemia in neonates and can lead to irreversible brain damage (1). CHI is characterized by the inappropriate over secretion of insulin. Usually, the diagnosis of CHI is established by confirming hypoglycemia (glucose <2 mmol/L), along with an inappropriately high insulin level (>2 mU/ml), a negative urinary ketone result, and normal tandem mass spectrometry findings (2). Histologically, CHI in neonates and infants can be divided into three types: diffuse, focal, and atypical. Gene mutation is regarded as the main cause of CHI. Genetic testing is helpful to differentiate the focal and diffuse forms of CHI and determine the surgical strategy if conservative medical treatment fails (3–5).

Patients with CHI are managed with frequent feeding or glucose infusion and using medications, such as diazoxide, octreotide, or glucagon, to control hypoglycemia. Conservative treatment is effective in approximately half of patients with CHI (6). For those in whom medical treatment fails, surgical intervention is required.

For diffuse CHI, near-total resection of the pancreas is an effective way to control symptoms, but for focal lesions, local resection of the lesion can achieve the goal of cure. There are reports in the literature of open surgery being used for CHI with focal lesions located in the head of the pancreas, but there have been no reports of laparoscopic surgery in this context yet. In this article, we reported our first two cases of successful laparoscopic treatment for local-type CHI with near-total pancreatic head resection and reconstruction of the remained body and tail of the pancreas and gastrointestinal tract through transmesocolic pancreaticojejunal Roux-en-Y loop anastomosis.

## Methods

### Study Population

Between July 2021 and November 2021, two patients (one boy and one girl, aged from 20 days to 1 month) with CHI who failed to respond to medical therapy were managed with laparoscopic pancreatic head resection and Roux-en-Y pancreaticojejunostomy in the Department of Pediatric Surgery, Guangzhou Women and Children's Medical Center. The surgical technical details and outcomes were analyzed. All procedures performed in the study involving human participants were in accordance with the ethics standards of the institutional research committee. Each patient's parents provided were written informed consent before the patient was enrolled in the study.

### Presentation

Patient 1 was a male infant with a normal spontaneous full-term delivery. Three days after birth, shortness of breath and foaming at the mouth were observed. The patient was admitted to the local hospital and diagnosed with hyperinsulinemia when his blood glucose was 2.21 mmol/L and his insulin was 11.3 μIU/ml. His blood glucose could not be maintained stably and was he then transferred to our center. Further examination of genetic test results suggested a heterozygous paternally inherited ABCC8 mutation [c.2113C>T(p.R705^*^)], which is usually suggestive of focal disease. Ultrasound examination showed normal findings for the pancreas and other parenchymal organs. 18-Fluoro-dihydroxyphenylalanine (18F-DOPA) positron emission tomography (PET)/computed tomography (CT) examination showed focal CHI in the head of the pancreas. After unsuccessful medical treatment, the patient underwent surgery at the age of 68 days and a body weight of 6.5 kg.

**Figure 1:**
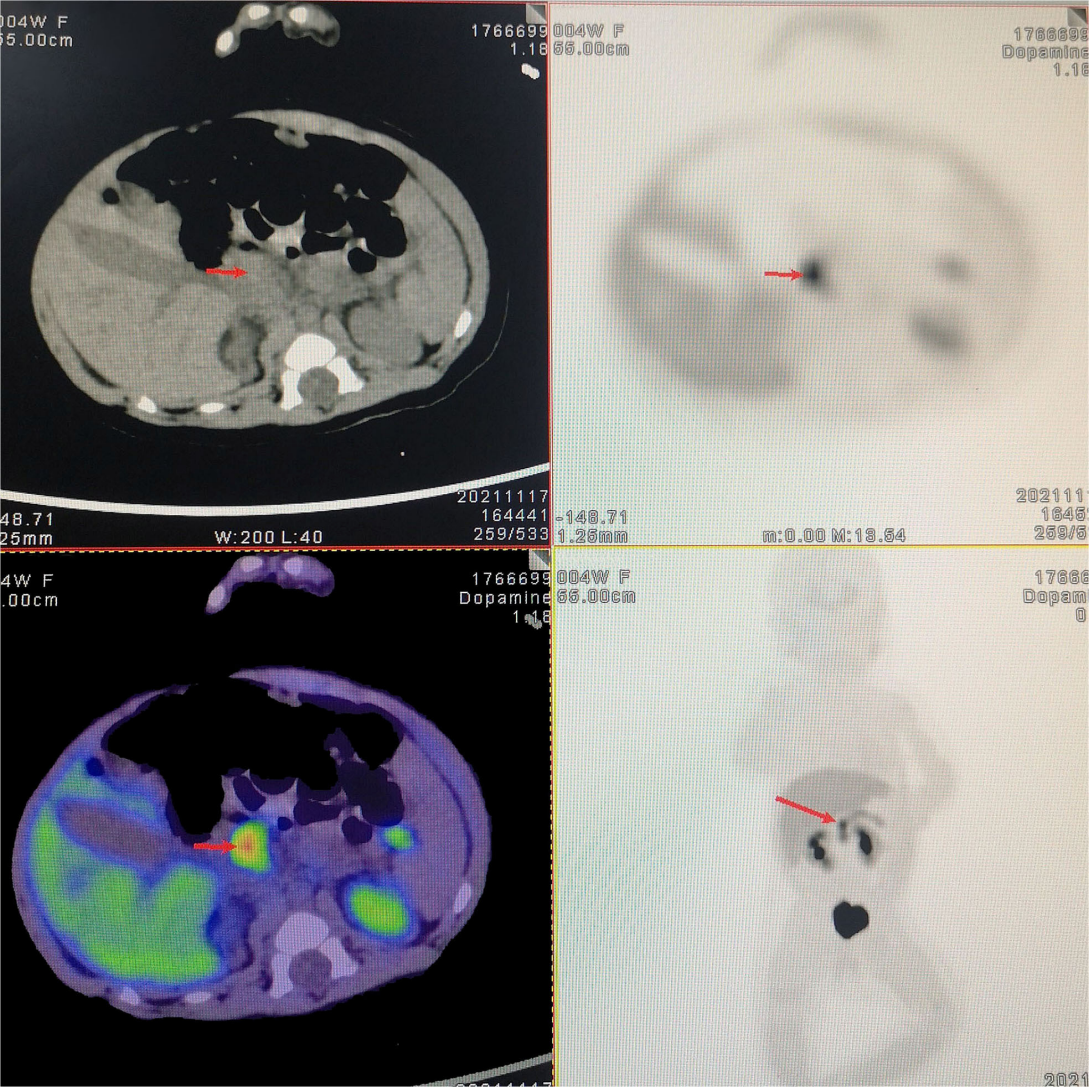
18-Fluoro-dihydroxyphenylalanine (18F-DOPA) positron emission tomography/computed tomography (PET/CT) scan. Preoperative 18F-DOPA PET/CT shows a focal lesion in the head of the pancreas.

Patient 2 was a 20-day-old female neonate whose mother was diagnosed with connective tissue disease and gestational diabetes mellitus. Sixteen hours after birth, she had episodes of cyanosis and hypoglycemia (2.1 mmol/L), and euglycemia had to be maintained by continuous intravenous glucose infusion. She was transferred to our center and the diagnosis of hyperinsulinemic hypoglycemia was established by the fasting test. The pancreatic sonographic findings were normal. Whole-exome sequencing identified a heterozygous mutation in the ABCC8 gene (p.Thr1042fs), which was inherited from her father. Further PET/CT scans demonstrated focally increased uptake of 18F-DOPA (0.6 × 0.7 cm) in the head of the pancreas (Figure 1). Diazoxide and octreotide administration was initiated but still failed to achieve normoglycemia. Therefore, laparoscopic near-total pancreatic head resection with Roux-en-Y pancreaticojejunostomy was performed at the age of 43 days and body weight of 5.2 kg.

### Surgical Technique

The surgical methods applied in the two cases were similar. The surgical procedure was as follows (a supplemental short operation video is available in Figshare, https://doi.org/10.6084/m9.figshare.20033057).

**Figure 2:**
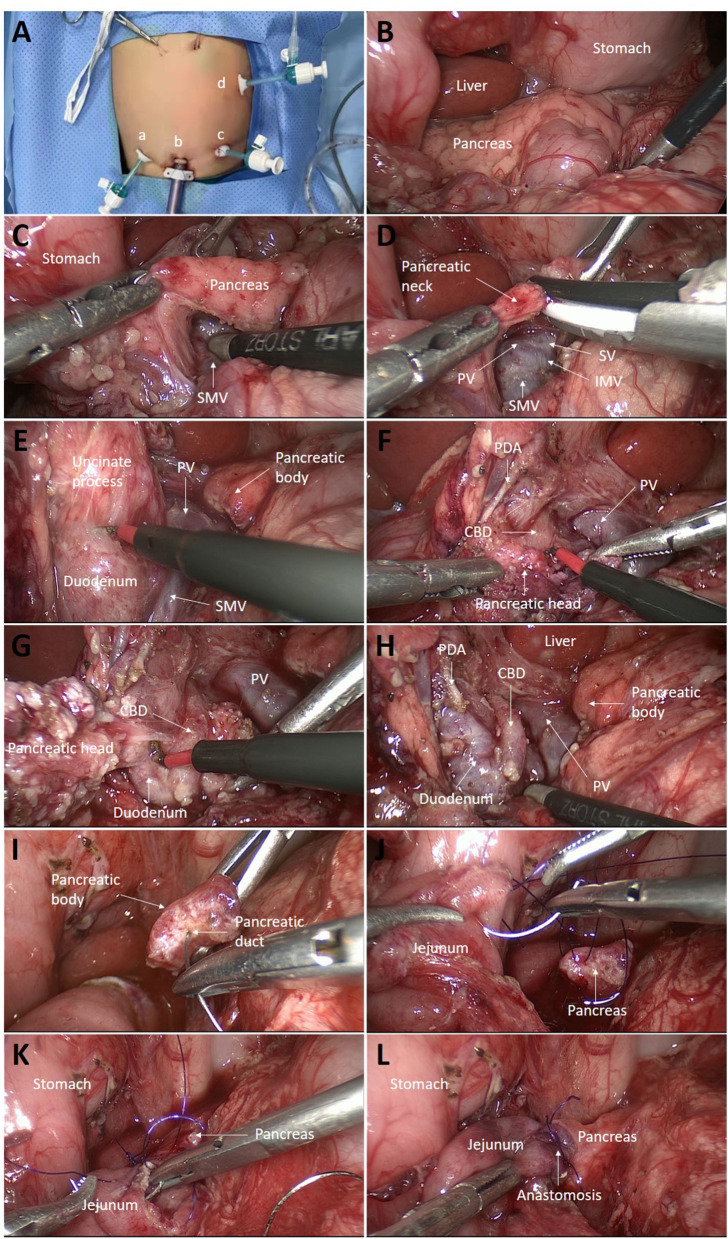
Intraoperative images. **(A)** Trocar locations: a, first auxiliary hole; b, lens hole; c, second auxiliary hole; and d, third auxiliary hole. **(B)** The pancreas was inspected for any localized lesions. **(C)** The post-pancreatic tunnel was created. **(D)** The pancreatic neck was transected with an ultrasonic scalpel. **(E)** The uncinate process was dissected along with the pancreatic capsule with electric cautery. **(F)** The superior margin of the pancreas was dissociated below the duodenal bulb, exposing the PDA and the CBD below the artery. **(G)** Pancreatic tissue surrounding the CBD was carefully removed bit by bit from top to bottom. **(H)** Near-total pancreatic head resection was completed, leaving only a small amount of pancreatic tissue around the PDA. **(I)** The pancreatic duct was verified by inserting the tail of a needle of 5-0 polydioxanone (PDS). **(J)** Roux-en-Y pancreaticojejunostomy was performed. First, the posterior wall of the jejunum and the pancreas was continuously sutured from the upper end of the jejunum, leaving the knot outside the intestinal wall. **(K)** The anterior wall of the jejunum was then continuously sutured. Finally, the two threads were tied at the lower end of the anastomosis with the knot outside. **(L)** The anastomosis was completed by enfolding the exposed pancreatic section in the jejunum. PDA, pancreaticoduodenal artery; CBD, common bile duct; SMV, superior mesenteric vein; IMV, inferior mesenteric vein; PV, portal vein; SV, splenic vein.

Under general anesthesia, the child was placed supine with the legs in a frog-like position at the lower end of the operating table, which was tilted in the reverse Trendelenburg position. The surgeon stayed at the lower end of the table with the camera person to his left and the assistant surgeon to his right. The screen was placed above the patient's head. The first port was established in an open manner through the inferior umbilical fold for a 5-mm 30° telescope. The CO_2_ pneumoperitoneum pressure was 8 mmHg, and the flow was 10 L/min. Two additional 3.5-mm ports were placed just at the level of the umbilicus approximately 5 cm to the left or right side of the umbilicus for 3-mm working instruments (Figure 2A). The fourth 3.5-mm port was inserted into the left upper abdomen.

The gastrocolic ligament was opened transversely followed by suspension of the posterior wall of the stomach with two transabdominal stay sutures to expose the lesser sac. Then, the pancreas was inspected for any localized lesions, specifically focusing on the pancreatic head, where PET/CT indicated a lesion (Figure 2B). These two patients did not show any signs of localized disease. Then, the tail of the pancreas was mobilized first by freeing it from the splenic hilum. A 5-mm biopsy was collected from the tail of the pancreas for fast-frozen section analysis. The results showed a normal pancreatic structure, with no abnormal islet cells. Therefore, it was decided to proceed with the planned laparoscopic pancreatic head resection and pancreaticojejunostomy.

The superior mesenteric vein and splenic vein were found along the middle colic vein. After dissecting the gap between the pancreas and the portal vein (Figure 2C), the pancreatic neck was cut with an ultrasonic scalpel just above the portal vein (Figure 2D).

The portal vein was pulled to the left, and the uncinate process was dissected along with the pancreatic capsule with electric cautery (Figure 2E). The branch vessels of the head of the pancreas into the portal vein were ligated. To fully expose the uncinate process, the Henle trunk was disconnected. The superior margin of the pancreas was dissociated below the duodenal bulb, exposing the pancreaticoduodenal artery and the common bile duct (CBD) below this artery (Figure 2F). The pancreatic tissue in front of the CBD was carefully cut bit by bit along with the CBD from top to bottom with electric cautery so that the front wall of the CBD was completely exposed until it entered the duodenum (Figure 2G). The loose adhesion between the CBD and the pancreas was separated, and the surrounding pancreatic tissue was removed, leaving only a tiny residual piece of pancreatic tissue between the CBD and the duodenal wall to protect the arterial supply of the CBD. Near-total pancreatectomy was completed by excising pancreatic tissue close to the inner part of the duodenum, leaving only a small amount of pancreatic tissue around the pancreaticoduodenal artery (Figure 2H).

**Figure 3:**
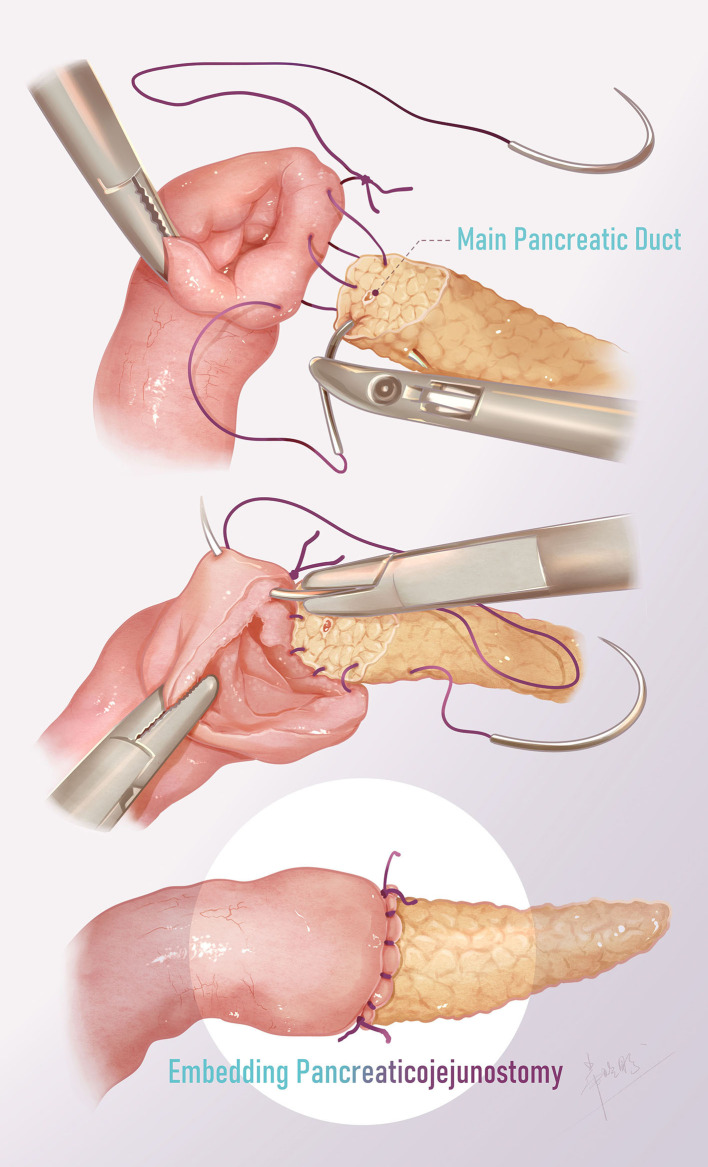
Surgical technique of pancreaticojejunostomy. The posterior and anterior walls were continuously sutured from the upper end of the anastomosis, and the two threads were finally tied at the lower end with the knot outside.

The end-to-side jejunojejunostomy was carried out extracorporeally by exteriorizing the proximal jejunum through the extending umbilical incision. Subsequently, the Roux jejunal limb was brought through the transverse mesocolon to the pancreatic body intracorporeally. Laparoscopic pancreaticojejunostomy was then performed (Figures 2I–L and 3). The pancreatic section was gently trimmed with scissors to remove the surface eschar and expose the pancreatic duct. The diameter of the pancreatic duct is small, but under the magnified view of the laparoscope, it can be identified by finding the small amount of flow of pancreatic fluid. Then, the pancreatic duct was verified by inserting the tail of a needle of 5-0 polydioxanone (PDS; Figure 2I). The end of a 25-cm-long Roux-en-Y jejunal limb was then pulled up through the mesocolon.

The ends of two 15-cm-long 5-0 monofilaments were tied together, and the two needles were used to suture the posterior and anterior walls respectively. First, one needle was inserted from the upper end of the jejunal anastomosis, leaving the knot outside the intestinal wall, and the posterior wall of the jejunum and the pancreas was continuously sutured (Figure 2J). Then, another thread was used to suture the anterior wall from the upper end of the jejunum (Figure 2K). Finally, the two threads were tied at the lower end of the anastomosis with the knot outside. The anastomosis was thus completed by enfolding the exposed pancreatic section in the jejunum (Figure 2L). The abdominal drain was placed just at the site of the anastomosis.

## Results

**Figure 4:**
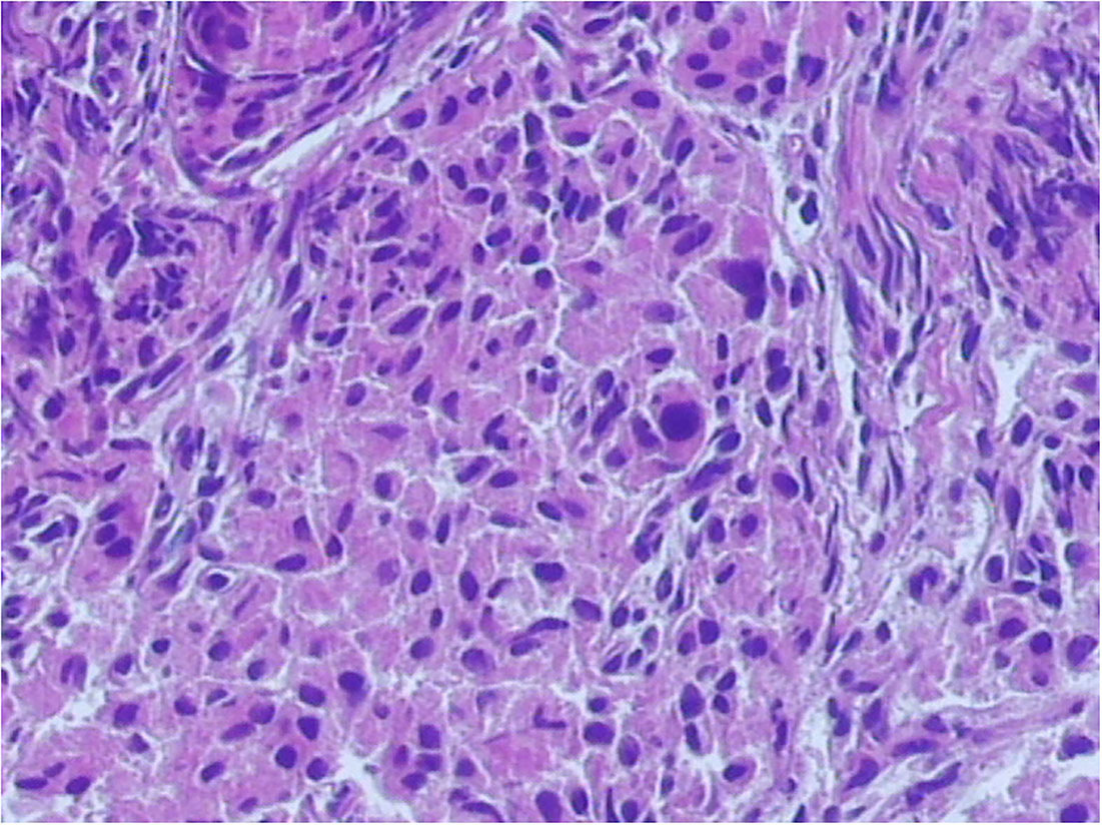
Histopathology section in high power view. Histological examination confirmed a diagnosis of focal congenital hyperinsulinism of infancy (CHI), characterized by the nodular aggregation of hypertrophic beta cells.

A laparoscopic procedure for near-total pancreatic head resection with Roux-en-Y pancreaticojejunostomy was successfully conducted in both patients. Neither patient required conversion to an open procedure. The operative duration was 300–330 min, and the intraoperative blood loss was approximately 10 ml in both procedures. Postoperative recovery was uneventful, with no cases of a biliary fistula or pancreatic leakage, and the abdominal drain was removed 4–5 days after surgery. Oral feeding commenced 3–4 days postoperatively, after the return of bowel activity. The blood glucose level was normalized gradually after the operation. Both patients were discharged 14 days postoperatively without any complications. Histological examination confirmed the diagnosis of focal CHI, characterized by hypertrophied beta cells with abnormally large nuclei (Figure 4). The blood glucose level remained normal under a normal diet over the 6-month follow-up period.

## Discussion

The surgical strategy and prognosis for diffuse- or focal-type CHI are quite different. Usually, a subtotal of 90–98% pancreatic resection is needed for diffuse-type CHI, but diabetes mellitus develops in 12–56% of patients, depending on the extent of surgery, age at surgery, and length of follow-up (7, 8). For patients with focal-type CHI, selective partial pancreatectomy is a curative surgical procedure (9, 10), and approximately 97% of patients were cured in a series of 246 cases of focal CHI reported by Adzick (9). Therefore, preoperative differentiation between the diffuse and focal types is critical for the treatment of CHI.

Usually, conventional imaging studies, such as ultrasound, CT, and magnetic resonance imaging (MRI), can only show nearly normal images of the pancreas and cannot be used to detect pancreatic lesions in patients with CHI (11). Genetic testing and PET/CT are regarded as reliable methods for distinguishing between the two types of CHI. The accuracy of 18F-DOPA PET/CT in locating pancreatic lesions can reach 100% (12–14); thus, it is helpful in formulating a precise surgical plan.

Superficial and small lesions in the pancreas can be treated by enucleation or simple resection. However, for deep pancreatic lesions, segmental resection, or limited pancreatectomy may be conducted. Local lesions can be cured after surgery. For lesions located at the head of the pancreas, surgery should include near-total resection of the head and reconstruction of pancreatic drainage (Roux-en-Y pancreaticojejunostomy). Open surgery is a mature technique for this procedure, but laparoscopic pancreatectomy has been previously reported only in central or distal pancreatectomy (2, 15, 16). Adzick reported the outcomes of a group of 59 CHI patients with lesions located at the head of the pancreas; 36 were amenable to enucleation, while 23 underwent total or near-total pancreatic head resection (two Whipple surgery). All patients were cured of CHI, and none developed diabetes or pancreatic exocrine insufficiency (17).

The technique of pancreatic head resection for focal lesions is similar to that for diffuse lesions. It is required to skeletonize the CBD and preserve a small amount of pancreatic tissue around the pancreaticoduodenal artery inside the duodenal loop (17). However, laparoscopic surgery is technically challenging. Although duodenal-preserving pancreatic head resection has been applied in surgery for benign lesions in adults (18), for neonates and infants, the small surgical space and small tissue structures make laparoscopic surgery quite difficult.

Adzick (9) believed that 98% of near-total resection was necessary for diffuse lesions to ensure surgical results and prevent a postoperative recurrence. In 98% of near-total resection, the CBD should be skeletonized, and only a tiny portion of the pancreatic head should be left between the CBD and the duodenal wall. Al-Shanafey (2) and Liem (16) reported 85–95% subtotal pancreatectomy for diffuse-form CHI by laparoscopy in 2009 and 2010, respectively. In the laparoscopic operation, the pancreas was removed, with 1 cm or more of pancreatic tissue along with the duodenum. Adzick (9) questioned the feasibility and effectiveness of laparoscopic surgery, arguing that instead of near-total resection, it should be called distal pancreatectomy (19). By comparing open surgery and laparoscopy in CHI, Al-Shanafey admitted that the extent of resection was significantly higher in the open group than in the laparoscopic group (10). CBD-related complications (intraoperative injury or postoperative stricture) have been reported to occur in up to 16% of pancreatectomies involving the pancreatic head (20, 21); thus, management of the CBD is one of the difficulties in laparoscopic pancreatic head resection in neonates and infants.

Histologically, focal CHI lesions are usually irregularly shaped and frequently have octopus-like tentacles; incomplete resection can lead to recurrence (17). Therefore, for local lesions of the head of the pancreas, near-total resection can prevent residual lesions and postoperative recurrence to the greatest extent. In the two cases in this study, the head of the pancreas was nearly completely resected, the CBD was skeletonized, and only a small amount of pancreatic tissue was preserved between the CBD and the inner part of the duodenum; additionally, the pancreatic and duodenal vasculature were not damaged.

There are several key points in the laparoscopic procedure. The identification and dissection of the CBD are the first one. In the laparoscopic procedure, the pancreaticoduodenal artery can be exposed after dissecting the upper margin of the pancreas, and the CBD can be observed below and behind it. The diameter of the CBD is approximately 2–2.5 mm, and it can be clearly identified under laparoscopic magnification. The CBD is enclosed in the pancreas, and its anterior wall can be exposed by opening the pancreatic tissue little by little with an electric knife. The dissection continues along with the CBD until reaching the duodenal wall, exposing the CBD completely. After dissecting the loose adhesion between the CBD and pancreas, the pancreatic tissue is removed, and the CBD is skeletonized.

Managing the uncinate process is another key point in laparoscopic pancreatic head resection. Part of the uncinate process passes behind the portal vein. An assistant is required to pull the portal vein to the left to expose the surgical field. Sometimes, the Henle trunk affects the exposure to the uncinate process; it can be severed during the operation to achieve full surgical field exposure. The amputation of the Henle trunk does not affect the blood supply to the second or third segment of the duodenum. One of the two patients in this study underwent amputation of the Henle trunk.

Pancreaticoenterostomy is the third key point of the laparoscopic operation. Zani (22) reported a series of 19 patients with CHI, three of whom required pancreatic head resection and pancreaticojejunostomy. All three patients underwent laparoscopic surgery, which was eventually converted to open surgery. To date, no cases of successful pancreaticojejunostomy in neonates or infants have been reported. To the best of our knowledge, this is the first report of such an operation performed by a laparoscopic approach.

The small pancreatic ducts in neonates and infants are difficult to recognize by the naked eye and sometimes need to be identified by intraoperative ultrasound during open surgery (3). In laparoscopic surgery, the eschar in the section of the pancreas is carefully cut with scissors. With laparoscopic magnification, pancreatic fluid overflow can be observed to determine the location of the pancreatic duct, which can be confirmed by inserting the needle tail of a 5-0 PDS suture into the pancreatic duct. During anastomosis, pancreatic duct damage should be avoided.

Traditional open pancreaticojejunostomy is performed using interrupted sutures to wrap the exposed section of the pancreas in the jejunum. In laparoscopic surgery, we modified the suture method to simplify the operation. Two 5-0 PDS stitches, each 15-cm long, were tied at the end of the suture. Starting from the external jejunal needle, the posterior wall and anterior wall of the anastomosis were continuously stitched from top to bottom and finally tied at the lower end of the anastomosis. In addition, when suturing the jejunum, the needle was introduced in an oblique manner to hitch more serosa and less mucosa, such that the serosal surface could be fully attached to the cut surface of the transected pancreatic body. In this way, the operation was simplified, laparoscopic surgery could be successfully completed, and anastomosis was ensured to avoid the occurrence of pancreatic leakage.

## Conclusion

To the best of our knowledge, this is the first report of successful laparoscopic near-total pancreatic head resection for focal pancreatic head CHI. In summary, we have demonstrated that laparoscopic pancreatic head resection with Roux-en-Y pancreaticojejunostomy is a safe and effective procedure in neonates and small infants with CHI.

## Data Availability Statement

The original contributions presented in the study are included in the article/supplementary material, further inquiries can be directed to the corresponding author/s.

## Ethics Statement

The studies involving human participants were reviewed and approved by the Ethics Committee of Guangzhou Women and Children's Medical Center. Written informed consent to participate in this study was provided by the participants' legal guardian/next of kin. Written informed consent was obtained from the individual(s), minor(s)' legal guardian/next of kin, and for the publication of any potentially identifiable images or data included in this article.

## Author Contributions

ZW, JW, QL, and WZ designed the study and drafted the initial manuscript. ZW, JW, XC, HN, and QH reviewed and revised the manuscript. All authors contributed to the article and approved the submitted version.

## Conflict of Interest

The authors declare that the research was conducted in the absence of any commercial or financial relationships that could be construed as a potential conflict of interest.

## Publisher's Note

All claims expressed in this article are solely those of the authors and do not necessarily represent those of their affiliated organizations, or those of the publisher, the editors and the reviewers. Any product that may be evaluated in this article, or claim that may be made by its manufacturer, is not guaranteed or endorsed by the publisher.
